# Films Based on a Blend of PVC with Copolymer of 3-Hydroxybutyrate with 3-Hydroxyhexanoate

**DOI:** 10.3390/polym12020270

**Published:** 2020-01-28

**Authors:** Evgeniy V. Belukhichev, Vera E. Sitnikova, Evgenia O. Samuylova, Mayya V. Uspenskaya, Daria M. Martynova

**Affiliations:** 1 Saint-Petersburg State Institute of Technology, 190013 St. Petersburg, Russia; RandomJWhite@yandex.ru; 2 Klöckner Pentaplast Rus, 195248 St. Petersburg, Russia; 3 Institute BioEngineering, ITMO University, Kronverkskiy Prospekt, 49, 197101 St. Petersburg, Russia; v.e.sitnikova@gmail.com (V.E.S.); samuilova.eo@itmo.ru (E.O.S.); daria.martynova@gmail.com (D.M.M.)

**Keywords:** polyvinyl chloride, polyhydroxybutyrate, polymer blends, polymer films, packaging

## Abstract

Polymeric packaging materials are one of the factors of environmental pollution. Reducing the environmental burden is possible by increasing the environmental friendliness of packaging materials. In this work, we study polymer films based on polyvinyl chloride (PVC) with a copolymer of 3-hydroxybutyrate with 3-hydroxyhexanoate P (3-GB) (3-GG) with different component ratios. The process of processing blends in the process of obtaining a packaging film is considered. The optical characteristics of the obtained films are determined. Thermal analysis of the obtained films was carried out using the differential scanning calorimetry (DSC), TGA, and thermomechanical analysis (TMA) methods. The degree of gelling of the resulting mixture was determined. It is shown that PHB has miscibility with PVC.

## 1. Introduction

Over the past fifteen years, the share of polymeric materials in the production of packaging for food, pharmaceuticals and consumer goods has grown from 17% up to 32%. It is worth noting that this upward trend in using the polymer materials in packaging is still positive, projected to reach 40% by 2025.

The main polymers for packaging are polyethylene (PE), polypropylene (PP), polyvinyl chloride (PVC), polystyrene (PS), and polyethylene terephthalate (PET). These materials have high barrier properties, transparency, strength, and inertness with respect to packaged products. Polymer materials, replacing paper and cardboard packaging, allowed us to increase the shelf life and the radius of transportation of most food and pharmaceutical products [1]. Polyethylene, polypropylene, and polyvinyl chloride represent about 60% of total plastic volume for household goods, medical equipment, leisure products, and other major applications [2].

However, in contrast to all the positive qualities of polymer packaging, the problem of recycling polymer waste arose. Currently, each person consumes approximately 0.3 kg of plastic per day. According to the United Nations Environment Agency, about 300 million tons of plastic are produced annually in the world [3], half of which are disposable items, mainly food packaging. Only 14% of this colossal amount is collected for re-processing, and only 9% is actually re-processed; 12% is burned with the release of toxic substances. The remaining almost 80% goes to landfill or, even worse, illegally dumped into the oceans [4]. According to the latest estimates, about 13 million tons of plastic waste falls into the World Ocean.

Currently, there are many research projects related to the production of biodegradable materials for the production of polymer packaging. At the moment, a rather extensive class of biodegradable polyesters has been developed, which include such polymers as polylactic acid, polycaprolactone, polybutylene succinate, polybutylene adipate, polyhydroxybutyrate, and its copolymers [5,6].

Despite the large number of biodegradable polymers developed, only polylactic acid and its blends [7,8] are widely used in the production of packaging materials. In recent articles on polymer packaging there are references to biodegradable polyethylene furanoate as a new material for the production of water bottles [9]. The widespread use of biodegradable materials in packaging production is halted by their low manufacturability and high price compared to base polymers.

For example, polyhydroxyalkanoates (PHAs) synthesized using *Alcaligenes eutrophus* bacteria cost about $16 per kilogram, which is 18 times more expensive than polypropylene. When the polymer is synthesized by *Escherichia coli*, the price drops down to $4 per kilogram, which is comparable to the prices of other biodegradable polymers [10], but still remains higher than the price of large-capacity polymers.

Polymer blending is a convenient route for the development of new polymeric materials, which combine the excellent properties of more than one existing polymer. This strategy is usually cheaper and less time-consuming than the development of new monomers and/or new polymerization routes, as the basis for entirely new polymeric materials [11].

A promising way to speed up the process of introducing biopolymers into the polymer packaging industry is to develop special blends of base and biodegradable polymers. This approach will allow us to obtain materials with competitive cost and more stable manufacturability of the process of their processing.

Polyvinyl chloride (PVC) is one of the most commonly used materials in the production of food packaging, it has good technical properties and high polarity, which ensures its high compatibility with a wide range of polymers. This polymer consists of 57% of chlorine, which the reserves are considered almost unlimited, and only by 42% of the fossil hydrocarbons [12], which significantly reduces the carbon footprint in its production, in contrast to other large-capacity polymers used in packaging [13].

Polyhydroxybutyrate (PHB) is an intracellular polyester belonging to the family of polyhydroxyalkanoates (PHA), which can be used as an alternative to petroleum-based plastics, since its structural properties are similar to polypropylene, and it has advantages such as biodegradability, biocompatibility, and the possibility of production from renewable carbon sources [14,15].

Poly 3-hydroxybutyrate (3HB) with 3-hydroxyhexanoate (3HH) (3HB-co-3HHx) possesses improved mechanical property and processability compared to P (3HB) and P (3HB-co-3HV). Co-polymerization of P (3HB) with the 3HHx monomer unit, which has a longer alkyl side chain avoids isodimorphism as the 3HB and 3HHx monomer units could not fit into the crystalline lattices of each other. As the 3HHx molar fraction was increased from 0 to 25 mol%, the crystallinity of P (3HB-co-3HHx) decreased from 60% to 18% [16].

The properties of these biological polymers are affected by the same fundamental principles as those of fossil-fuel derived polyolefins, with a broad range of compositions available based on the incorporation of different monomers into the PHA polymer structure, and with this broad range tailoring subsequent properties [17].

This article describes the study polymer film on the base blend of polyvinyl chloride with a copolymer of polyhydroxybutyrate and polyhydroxyhexanoate.

## 2. Materials and Methods

### 2.1. Materials

The following materials were used:

- PVC suspension, *K_f_* = 58 (Shintech Inc., Houston, TX, USA);
- Organotin thermostabilizer based on dioctyltin bis (2-ethylhexyl thioglycolate) (Galata Chemicals, Southbury, CT, USA);
- Glycerol ester as an internal lubricant (Emery Oleochemicals, Telok Panglima Garang, Malaysia);
- Oxidized polyethylene wax as an external lubricant (Honeywell, Charlotte, NC, USA);
- A copolymer of 3HB with 3HH, the number average molecular weight of 500,000–600,000 daltons, polydispersity 2.3, the ratio of 3HB/3HH = 95/5 (Kaneka, Takasago, Japan).

All components used in this study was supplied Klöckner Pentaplast Rus Ldt (Saint Petersburg, Russian Federation). All reagents were used as is without further purification.

The basic characteristics of the polymers used are given in Table 1.

### 2.2. Preparation of Composition

Five blends were prepared, designated as: PVC/PHB0, PVC/PHB10, PVC/PHB20, and PVC/PHB30, with different ratios of PVC to PHB. The composition of the blends is shown in Table 2.

All blends were mixed in a high-speed HENSCHEL mixer (Kassel, Germany) at a temperature of 80 °C. Plasticization of the material was performed in a BUSS single-screw oscillating extruder (Pratteln, Switzerland) at a temperature of 180 °C and a rotation speed of 140 rpm. Films with a thickness of 500 μm (±5 μm) were made from the obtained melt by the roller method.

### 2.3. Methods

#### 2.3.1. Turbidity Determination

Turbidity was determined on a turbidity meter Diffusion Systems Ltd. M57 Spherical Hazemeter (BYK Gardner, Geretsried, Germany) by measuring the amount of light scattered as it passes through a film with a resolution of 0.1% according to ASTM D1003-13 Standard Test Method for Haze and Luminous Transmittance of Transparent Plastics hazemeters. For this, a sample with a minimum size of 25.4 mm was placed in a light-tight compartment for samples with 0 degrees illumination with diffuse viewing.

#### 2.3.2. Color Determination

The color parameters of the film samples are presented in the CIELab color space (also known as CIE L*a*b* or sometimes abbreviated as simply “Lab” color space) and determined using a BYK Gardner spectrophotometer (Geretsried, Germany).

Yellowness index (YI) per ASTM Method D1925 is calculated as follows:

(1) $$YI\;D1925=\frac{100\left(1.274976795X-1.058398178Z\right)}{Y}$$

Under C/2° conditions for all instruments except UltraScan XE.

Transformation of L, a, b (CIELab) data to X, Y, Z (CIE XYZ tristimulus) is most easily expressed using the inverse of the function *f* above:

(2) $$X=X_{n}f^{-1}\left(\frac{L^{*}+16}{116}+\frac{a^{*}}{500}\right)\;Y=Y_{n}f^{-1}\left(\frac{L^{*}+16}{116}\right)\;Z=Z_{n}f^{-1}\left(\frac{L^{*}+16}{116}-\frac{b^{*}}{200}\right)$$

where

(3) $$ff^{-1}\left(t\right)=\{\begin{array}{ll} t^{3} & if\;t>\delta \\ 3\delta^{2}\left(t-\frac{4}{29}\right) & otherwise \end{array}$$

where *δ* = 6/29.

#### 2.3.3. Gloss

The surface reflectance (gloss) of the films was determined using BYK-Gardner (Geretsried, Germany) SC-4510 Gloss Meter, High Gloss, 20° (ASTM D523, D2457). A gloss meter is an instrument that is used to measure specular reflection gloss of a surface. Gloss is determined by projecting a beam of light at a fixed intensity and angle onto a surface and measuring the amount of reflected light at an equal but opposite angle.

#### 2.3.4. Thermal Stability

Thermal stability was determined by determining the residual efficiency of the thermostabilizer. The method implies thermostating of samples of polymer films at a given temperature and loading speed. A sample film of 240 mm long and 10 mm wide is gradually introduced into the heating zone. As a result, we get a sample that has undergone thermal degradation under controlled conditions. The test results are presented in the form of a graph of the yellowing index (YI, %).

**Figure 1:**
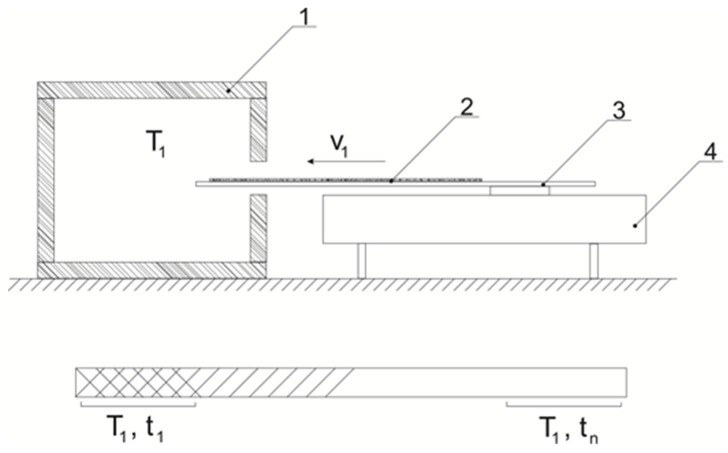
Schematic diagram of the installation for measuring the residual content of the thermal stabilizer: 1—thermostat, 2—sample film, 3—substrate for loading the sample, 4—engine; below, a schematic representation of the sample after the test; *T*_1_—test temperature; *V*_1_—sample loading speed; t_1_, *t*n is the residence time of the sample in the heating zone.

Schematic diagram of the installation for research and a schematic image of the sample are shown at Figure 1.

#### 2.3.5. Differential Scanning Calorimetry, DSC

The thermal properties of the obtained samples were studied on a differential scanning calorimetry (DSC) 204 F1 Phoenix differential scanning calorimeter (Netzsch, Selb, Germany) operating at a heating rate of 10 °C/min in the range from −30 to 250 °C. After the equilibration phase at 25 °C, the samples were first heated to 175 °C, then cooled to −30 °C, and then again heated to a temperature of 250 °C. Nitrogen was used as purge gas at a flow rate of 40 mL/min. The weight of the film samples used for DSC measurements ranged from 5.5 to 8.5 mg. Samples were placed in aluminum crucibles with a punched cap. The melting temperature (*T_m_*) and apparent melting enthalpy (*H_f_*) of each sample were determined from the maximum and the area of the endothermic melting peaks during the first scan, respectively. The data of the second heating curve were analyzed to determine the glass transition temperature *T_g_*.

#### 2.3.6. Thermomechanical Analysis, TMA

Thermomechanical analysis (TMA) experiments were carried out on a TMA 402 F1 Hyperion (Netzsch, Selb, Germany) operating in the penetration mode. Samples were heated from 25 to 175 °C with a heating rate of 5 K/min. Nitrogen was used as purge gas at a flow rate of 40 mL/min. The glass transition temperature was determined by changing the slope of the obtained curves.

#### 2.3.7. Thermal Gravimetric Analysis, TGA

Thermogravimetric analysis was used to study the thermal stability of the samples. All samples were measured using a Libra 209 F1 thermogravimetric analyzer (Netzsch, Selb, Germany) with a scan rate of 10 °C/min in a nitrogen atmosphere in the temperature range of 25–900 °C.

## 3. Results

**Figure 2:**
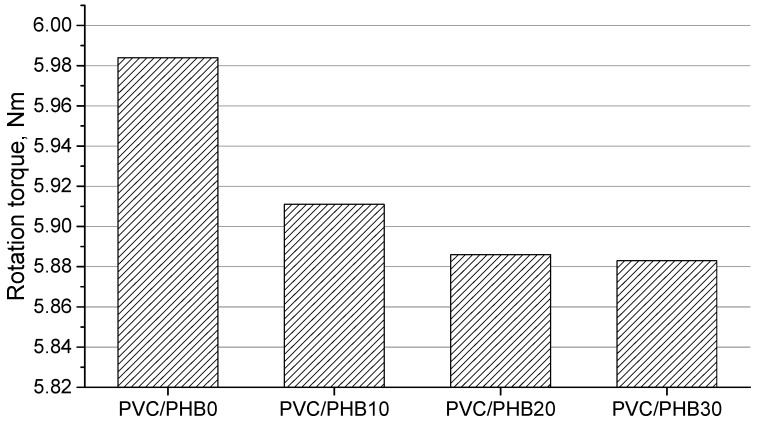
The values of the rotation torque in the extruder in the processing of PVC/PHB blends.

The dependence of the extruder torque during the processing of blends PVC/PHB on the increase in the proportion of PHB is shown on Figure 2. The graph shows that an increase in the proportion of PHB in the blends leads to a decrease in the torque of the extruder. All blends containing PHB showed the effect of accelerating the shear melting of PVC particles and greater melt elasticity, which shows the effectiveness of PHB as a processing additive [21]. It should be noted that elastomers based on nitrile butadiene rubber and ethylene vinyl acetate copolymer, which are solid polymer plasticizers, have a similar effect on the processing of polyvinyl chloride [18]. When processing on the extruder, significant fluctuations in the flow of the melt were not detected.

With further processing on the rollers, increased adhesion to the metal surface of the shafts caused by the presence of PHB was observed. The presence of such adhesion can also be a factor in accelerating the process of melting the blend in the extruder.

The separating force of the melt in the gap in the presence of PHB decreases, which usually has a positive effect on the calendaring process. Lower spacer pressure allows one to reduce the load on the calendar equipment necessary for resistance to melt pressure [22] (roll bending and cross-axis), and to obtain a film with smaller thickness differences across the width of the canvas.

The optical properties of the polymer blends used in the production of packaging materials determine the range of products that can be obtained using these blends. Films having a high haze and a dull surface are most often not used for the production of packaging materials.

Table 3 shows the results of measuring the optical characteristics of the films obtained from PVC/PHB blends with different ratios of these polymers. It was noted that an increase in the PHB content in the blend leads to less light scattering in the visible range (400–700 nm) passing through the thickness of the obtained films. The low optical haze (high transparency) of the samples can be associated with good compatibility of these polymers [23], since films from blends of incompatible polymers are characterized by high turbidity due to differences in polymers refractive indices [24].

High values of gloss on the surface of the films obtained from blends of PVC/PHB20 and PVC/PHB30 indicate uniform mixing of the components of the blend and a low number of surface defects, which in turn can also indicate the compatibility of the tested polymers in the melt.

The color of the obtained samples varied slightly. An increase in the values of channel L for transparent films was most often associated with a decrease in turbidity. An increase in the value of channel b during the study of PVC films was most often associated with the formation of chromophore groups during polymer degradation, but in this case, in the absence of significant changes in channel a, it might relate to an increase in the fraction of PHB in the system, which has its own yellow tint.

**Figure 3:**
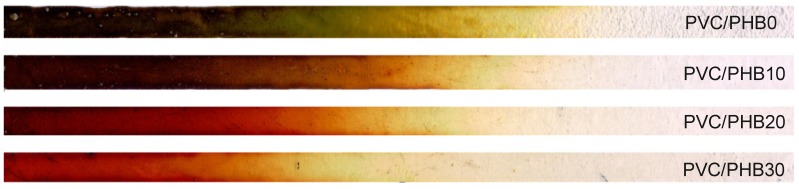
The appearance of the samples after the test for the effectiveness of the residual stabilizer at 220 °C.

**Figure 4:**
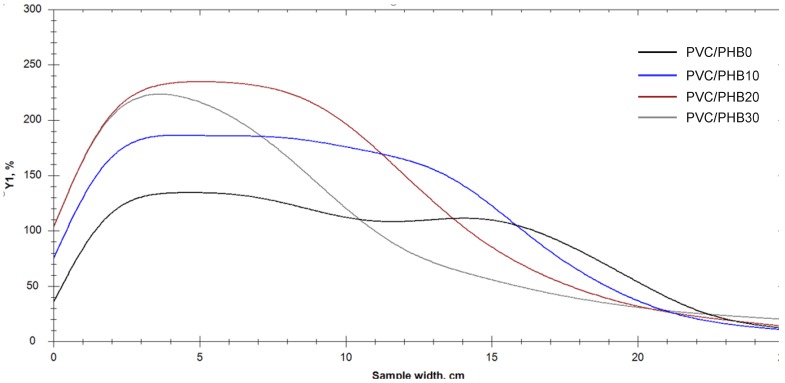
Graphs of yellowing index change (YI, %) for tested samples.

According to the results of the thermal stability test by determining the effectiveness of the residual stabilizer, we could conclude that an increase in the proportion of PHB in the PVC-PHB system had a positive effect on the duration of thermal stability of the obtained films. Test results are shown in Figure 3 and Figure 4.

This positive effect was most likely due to a simple decrease in the fraction of easily degrading polyvinyl chloride in the PVC-PHB system, since it is difficult to assume that high molecular weight polyhydroxybutyrate can have a stabilizing effect.

However, a decrease in the melt viscosity upon introduction of PHB slowed down the processes of thermo-mechanical destruction during processing by extrusion followed by calendaring, which was reflected in the initial color values and partially affects the increase in the duration of the thermal stability of the blend.

Determining the thermal stability of PVC is very important because of its thermal and shear sensitivity during processing. This parameter provides valuable information about processing conditions, including when PVC serves as a matrix of composites with natural fillers [25].

**Figure 5:**
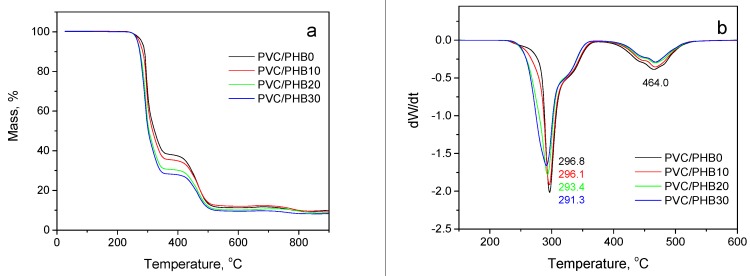
Thermogravimetry (TG; **a**) and derivative thermogravimetry (DTG; **b**) curves of PVC-based polymer films with different PHB contents.

Usually, two stages of degradation are observed in PVC: the first stage is the removal of hydrogen chloride along with some small volatile molecules of saturated and unsaturated aliphatic or aromatic hydrocarbons. The second stage involves the formation and volatilization of intramolecular cyclization products. In unfilled PVC, the first stage of decomposition proceeded at about 297 °C, the second, about 464 °C (Figure 5).

Polyhydroxybutyrate, as well as PVC, is sensitive to thermal decomposition (Figure 5a, curve 5). It is known that at high temperatures (above 200 °C) PHB thermally degrades with the formation of γ-butyrolactone (cyclic monomer). At higher decomposition temperatures, other acid molecules may form, such as oligomeric acid crotonates, which upon further degradation form low-molecular acids (mainly crotonic and 3-butenes) [26].

Although the degradation of PVC and PVC/PHB films was consistent with similar trends, there were differences in the shape of the TG curves, especially in the range 260–360 °C (Figure 5b). It was especially clearly seen that the 50%-weight loss temperature shifted toward lower temperatures with increasing concentration of PHB in the PVC/PHB films. The temperatures of 1%, 5%, 10%, and 50%—weight loss of all PVC samples are shown in Table 4.

These data show that temperatures with losses of 1%, 5%, 10%, and 50% associated with progressive degradation are lower for composites containing polyhydroxybutyrate than for unfilled PVC. Modification of PVC using a biopolymer led to a decrease in the thermal stability of the polymer, which decreased significantly with increasing PHB content in the PVC matrix.

**Figure 6:**
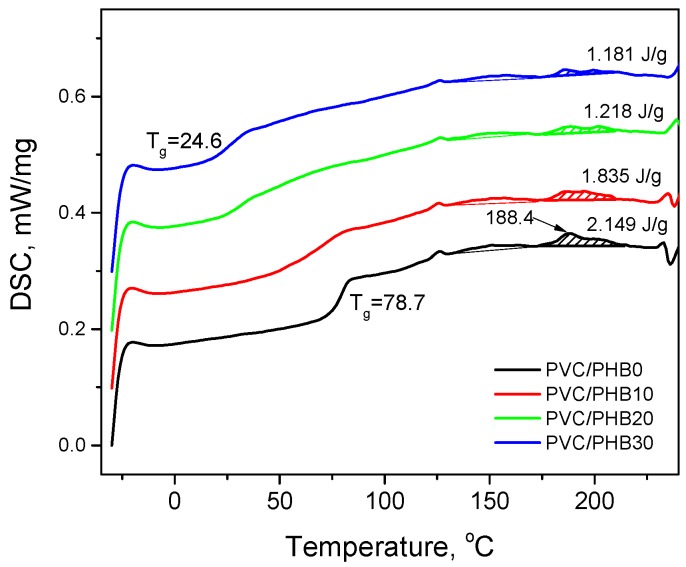
Differential scanning calorimetry (DSC) curves of the second heating of PVC/PHB films of various compositions.

Important properties regarding the thermal stability of PVC/PGB films were obtained by differential scanning calorimetry (DSC). Figure 6 shows the DSC curves for the second heating of the PVC/PHB films, since after the first heating the curves show an endothermic peak directly above the *T_g_* of the sample, which corresponds to the enthalpy relaxation experienced by the PVC in the film.

Glass transition temperatures of 78.7 and 0.1 °C were characteristic of pure PVC and PHB, respectively. On the DSC thermograms obtained for all PVC/PHB films, the glass transition temperatures characteristic of the pure components was absent; instead, there was a single glass transition that depended on the blend composition. The formation of a single-phase system with one *T_g_* serves as an indicator of the miscibility of components in blends [27,28]. However, a film from blend of PVC with 10% PHB has a wide transition from a glassy state, which indicates some chemical heterogeneity. In addition, on the DSC curves of PVC/PHB films, there were no melting peaks characteristic of semi-crystalline PHB, which were at temperatures of 126 and 140 °C.

**Figure 7:**
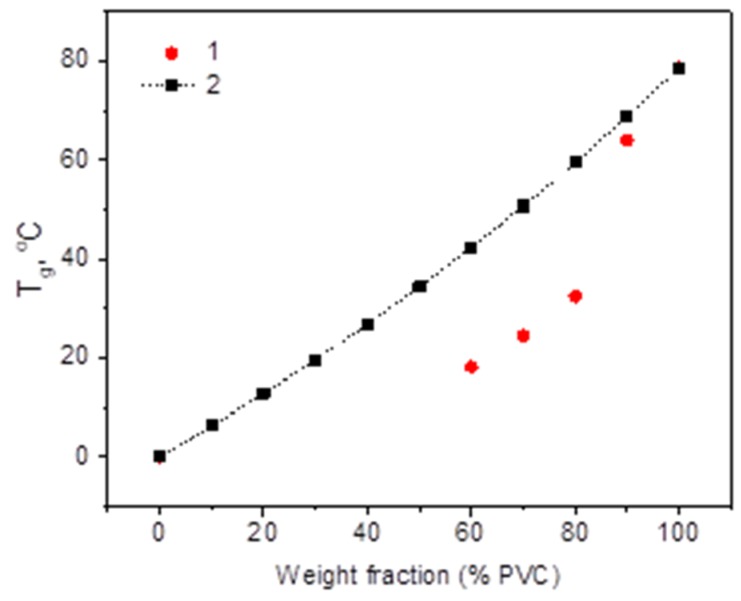
*T_g_* change in PVC/PHB blends: 1—experimental data and 2—*T_g_* from Fox eq.

DSC analysis confirmed the formation of a single *T*_g_-transformation, which shifted in the expected direction. The glass transition temperatures in PVC/PHB films (Figure 7) show a negative deviation from the calculated Fox addition law (4). A negative deviation is an indicator of the miscibility of the components in the blend [29]:

1/*T*_g,b_ = w_1_/*T*_g1_ + w_2_/*T*_g2_ (4)

where *T*_g,b_ is the glass transition temperature of the films from blend of polymers, *T*_g1_ and *T*_g2_ are the glass transition temperatures of pure polymers that make up the blend, w_1_ and w_2_ are the specific gravity in the blend of the first and second components, respectively.

PGB, which had good miscibility with PVC and a low glass transition temperature, was expected to act as a high molecular weight plasticizer, protecting the strong dipole binding forces responsible for the stiffness of the PVC chains and providing more free volume, which in general would lead to increased stability, mobility, and flexibility of PVC chains.

An important parameter in the processing of PVC-containing blends is the gelation degree (G, %), which characterizes the final morphology of the system depending on the processing conditions [30]. After the polymerization stage, PVC exhibited a complex morphology consisting of a hierarchy of domains of different sizes. The smallest domains (microdomains) contained ordered or crystalline regions, which accounted for approximately no more than 10% of the weight of PVC. PVC was characterized by DSC curves, which had a wide endothermic peak in the range from 140 to 230 °C.

At standard PVC processing temperatures (between 185 and 205 °C), melting remains partial. This molten crystalline portion will subsequently recrystallize during cooling, and newly created ordered objects are referred to as secondary crystallites. Thus, recycled PVC contains two types of crystallites, and the proportion of primary and secondary depends on processing and, mainly, on heating conditions [30].

The gelation process also affects the microstructure of PVC on a wider scale: during processing, the hierarchical structure of PVC is violated to a certain extent depending on the conditions of shear and heating.

Using the DSC technique for PVC compositions, two endothermic peaks are identified. Peak B (high temperature) is identified as melting of primary crystallites, and peak A (low temperature) is identified as melting of secondary crystallites. Using the following relation (5), including A and B enthalpies, it is possible to calculate the gelation level of PVC [31]:

*G* = *H_A_*/(*H_A_* + *H_B_*) ∗ 100 (5)

The gelation of PVC plays a significant role in strength, elastic modulus, tensile strength, and elongation, among other properties [32].

The calculation of the area under these two peaks was carried out for DSC curves of the second heating (Table 5). Figure 6 also shows that the area under the peak at a temperature of 188.4 °C (peak B, primary crystallites) decreases with an increase in the proportion of PHB in the blend, which indicates a decrease in the crystalline phase of PVC in the studied films.

The study results show that the introduction of PHB into the system had a significant effect on the level of gelation, which positively affects the optical properties of the resulting films.

**Figure 8:**
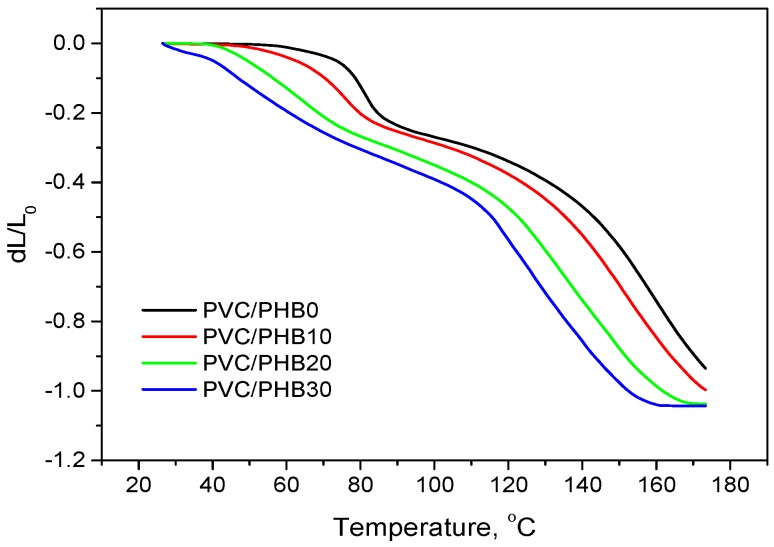
Thermomechanical analysis (TMA) curves of PVC/PHB blends with different PHB contents.

Thermomechanical analysis was carried out in order to track the dependence of the polymer deformation on temperature with its continuous increase. The data obtained during thermomechanical analysis of polymer films allow one to specify the temperature regimes of processing (processing window), restrictions for post-processing of polymer products (embossing, coating, grinding, etc.) and indicate indirectly the compatibility of polymers in the blend. The data obtained during thermomechanical analysis are shown in Figure 8 and Table 6.

It thus could be concluded that with an increase in the proportion of PHB in the PVC/PHB films, the softening temperature and yield temperature uniformly deviated to a range of lower values, which also characterizes the compatibility of PVC with PHB in the melt.

The thermomechanical curve became smoother, which indicates the expansion of the window for processing blends with a high proportion of PHB content and so the possibility of plasticizing these blends at lower temperatures, which, in turn, would positively affect the thermal and thermomechanical stability of the material, and reduce the cost of heating energy system.

## 4. Discussion

Summing up the results of study performed for the PVC/PHB blends, we concluded that this composition allowed one to get hard films by calendaring for further thermoforming. Processing the PVC/PHB blend may take place at lower temperatures, which not only reduces the energy consumption for heating the equipment, but also allows us to reduce the cooling time of the material, which will allow us to process this blend with higher productivity.

The resulting films had high optical characteristics (transparency, gloss, and initial color), most often necessary to ensure visual quality control of packaged food and pharmaceutical products by the end user.

One of the most important parameters of this blend is the increase of the environmental friendliness of its products [26]. This is due to the fact that it is possible to replace part of the environmentally harmful PVC in the final product (for example, in a packaging film) with polyhydroxybutyrate without deterioration of such parameters as turbidity, color, and gloss. In this case, the processing temperature of the mixture also decreased. This was due to the fact that, unlike PVC, the raw material for the synthesis of PHB is biomass (lignocellulose, starch, sucrose, vegetable oils, etc.), i.e., a renewable source implying the absorption of carbon dioxide, and therefore an increase in the proportion of PHB in the PVC/PHB system would reduce the carbon footprint of the final packaging material.

## Figures and Tables

**Table 1:** The basic characteristics of the tested polymers [18,19].

Properties	PVC	PHB
Density, g/cm^3^	1.37–1.43; 1.53 (crystalline material), 1.373 (amorphous material)	1.17–1.25; 1.262 (crystalline material), 1.177 (amorphous material)
Proportion of crystals, %	4–10	30–80
Cell type (lattice)	orthorhombic	orthorhombic
Freezing point, °C	82–87	−4–2.5
Melting point, °C	103–230	166–185

**Table 2:** Tested polymer compositions [20].

Sample	PVC, %	PHB, %	Stabilizer, %	Internal Lubricant, %	External Lubricant, %
PVC/PHB0	98.4	0	1.0	0.5	0.1
PVC/PHB10	88.4	10	1.0	0.5	0.1
PVC/PHB20	78.4	20	1.0	0.5	0.1
PVC/PHB30	68.4	30	1.0	0.5	0.1

**Table 3:** Optical characteristics of films obtained from PVC/PHB blends.

Optical Characteristics	PVC/PHB0	PVC/PHB10	PVC/PHB20	PVC/PHB30
Turbidity, %	46.6	24.7	3.5	4.8
Gloss (geometry 60°)	12.1	39.5	108.4	102.1
Color:				
L	86.78	88.74	89.89	88.74
a	0.49	0.42	0.33	0.35
b	0.12	0.20	0.77	1.91

**Table 4:** Thermal stability of PVC/PHB blends.

Sample	Temperature Value for Composite Weight Loss (°C)			
	1%	5%	10%	50%
PVC/PHB0	254.20	279.48	289.70	323.15
PVC/PHB10	252.63	272.25	283.38	316.53
PVC/PHB20	252.23	267.22	274.91	301.66
PVC/PHB30	253.04	266.81	273.44	301.10

**Table 5:** The level of gelation for PVC/PHB films.

Sample	*T_g_*, °C	*H_A_*, J/g	*H_B_*, J/g	*G*, %
PVC/PHB0	78.7	0.9961	2.149	31.67
PVC/PHB10	64.1	1.1160	1.835	37.81
PVC/PHB20	32.5	0.8683	1.218	41.62
PVC/PHB30	24.6	0.8845	1.181	42.82

**Table 6:** Thermomechanical analysis results for tested PVC/PHB blends.

Sample	Softening Point, °C	Flow Temperature, °C
PVC/PHB0	75.7	145.2
PVC/PHB10	65.7	136.6
PVC/PHB20	42.8	119.7
PVC/PHB30	40.1	112.2
